# Stakes of Knowing the Truth: A Motivational Perspective on the Popularity of a Controversial Scientific Theory

**DOI:** 10.3389/fpsyg.2021.708751

**Published:** 2021-09-09

**Authors:** Tiffany Morisseau, T. Y. Branch, Gloria Origgi

**Affiliations:** ^1^Laboratoire de Psychologie Appliquée et d'ergonomie, Institut de Psychologie, Université Paris Descartes, Boulogne-Billancourt, France; ^2^Strane Innovation, Gif-sur-Yvette, France; ^3^Institut Jean Nicod, ENS, PSL University, CNRS, Paris, France

**Keywords:** beliefs about science, cognitive accuracy, motivated reasoning, anti-science attitudes, social cognition, hydroxychloroquine, didier raoult, Covid-19

## Abstract

The aim of this article is to provide a different perspective on people's beliefs regarding controversial scientific information. We emphasize that, although people generally aim at getting a fair representation of reality, accuracy about scientific issues only matters to the extent that individuals perceive it as useful to achieve their own goals. This has important consequences in terms of how anti-science attitudes as well as epistemically questionable beliefs must be interpreted, which has consequences for addressing misinformation. We argue that most people who endorse scientific misinformation are not truly interested in its accuracy, and rather that plausibility at face value often suffices when it is meant to be used for social purposes only. We illustrate this view with the example of hydroxychloroquine, which was considered as potential treatment for Covid-19, and which has been the subject of much media hype and public concern, particularly in France.

## 1. Introduction

The question of beliefs about science is an eminently important one, which can be thought to have definite social and political consequences. Rather than stressing the importance of developing public education and media literacy, which is obviously an important endeavor, we propose a new perspective by showing how adherence to baseless scientific theories can be explained by something other than lack of scientific knowledge or critical thinking, namely a lesser epistemic interest in the truth of a matter. Questioning the relative importance of truth to people who share controversial ideas opens new avenues of research on how to remedy the negative consequences of the spread of these ideas at the societal level. In what follows, we revisit and analyse the case of public support for hydroxychloroquine as a treatment against Covid-19.

In late 2019, the outbreak of a new zoonotic coronavirus (SARS-CoV-2), causing severe acute respiratory syndrome (Coronavirus disease 2019 aka. Covid-19) would go on to become a global pandemic (WHO, 2020). In response, researchers immediately began speculating about potential solutions. Among the most promising candidates was a common drug called hydroxychloroquine (WHO Solidarity Trial Consortium, 2021). Listed as an essential medicine by the World Health Organization (WHO), hydroxychloroquine derives from chloroquine (which comes from quinine) and has been used to treat malaria for decades (Solomon and Lee, 2009). Hydroxychloroquine (branded by Sanofi Aventis as Plaquenil, hereafter HCQ), is a less toxic version of chloroquine, which is used to treat autoimmune conditions such as rheumatoid arthritis and lupus erythematosus.

The HCQ solution had two major advantages: availability and affordability. Moreover, as HCQ had been a well-known cure for other diseases, and for quite a long time, the innocuous nature of the drug could be taken for granted. These reasons quickly made it one of the most appealing leads in fight against Covid-19. In France, microbiologist Didier Raoult and his team at the IHU Méditerranée Infection Institute presented the positive results of a study on the effect of HCQ on Covid-19 in March 2020 (Gautret et al., 2020). This research would go on to raise ethical concerns^1^ and be criticized for being methodologically weak (Bik, 2020). Raoult's research raised the argument that the use of HCQ could potentially save many patients, even invoking the Hippocratic Oath (Sauvayre, 2020).^2^ Although relatively unknown to the general public a few months earlier, Raoult's reputation came under public scrutiny as his theorizing became increasingly popular, and he appeared to rank high in many indicators of trustworthiness (Branch et al., 2021). The interest was temporarily backed up by promising *in vitro* studies from China (Liu et al., 2020). The WHO and the European Medicines Agency thus recommended the use of HCQ as part of clinical trials or national emergency use programmes (EMA, 2020). Institutions such as the University Hospitals of Geneva (HUG) relied on it at the beginning of the pandemic, before abandoning it a few months later (Agoritsas et al., 2020).

In the weeks and months that followed, numerous studies challenged the assumption that HCQ was actually useful in the treatment of Covid-19 patients, and there is now a scientific consensus as to the lack of its efficacy (Lamontagne et al., 2021). However, arguments against HCQ relied on the absence of obvious beneficial effects in an increasing number of studies, rather than a single conclusive argument proving that it was harmful. HCQ therefore remained supported by many individuals in France and abroad, despite the extensive scientific data showing its lack of efficacy. A number of people, including politicians, further declared publicly that they had been successfully treated with HCQ (Sciama, 2020), even if this outcome could be more parsimoniously explained by spontaneous recovery.

## 2. Intuitive Framings

In an emergency situation, the urge to act is overwhelming. It seems morally preferable to do something rather than nothing, especially if we do not have much to lose. Raoult's argument further centered on the idea that researchers and data analysts were disconnected from the field, and that the methods they usually relied on (e.g., randomized controlled trials) could no longer be applied in a situation of crisis (Risch, 2020). Although it was inaccurate to say that randomized trials could be dispensed with when determining the actual efficacy of a drug, it surely made sense that the situation called for pragmatism. From the public's point of view, the “scientist-practitioner” narrative of adopting a pragmatic stance and saving lives certainly had every reason to be accepted. And HCQ was indeed one of the serious leads in international trials. The question is why subsequent studies invalidating this first legitimate intuition did not result in a weakening of belief in this solution, which was less and less likely to be effective.

Critically, it is one thing to consider a hypothesis as plausible—that is, compatible with one's priors—and another to assess the likelihood of that hypothesis being correct. For instance, the proposition p “HCQ reduces mortality in Covid-19 patients” is plausible if one's priors include the fact that it is recommended by a recognized expert, and that it is cheap and apparently safe at the individual level. Holding the effectiveness of an action to be true based on its mere plausibility can have positive outcomes (Damisch et al., 2010). It can be related to superstition, which is found in most if not all human cultures. Superstition has been interpreted as an adaptive tendency to favor type I over type II errors when making causal associations, in situations where falsely detecting the presence of a causal link has fewer negative consequences than missing it if it turns out to be true (Shermer, 1997; Beck and Forstmeier, 2007; Foster and Kokko, 2009). Refusing to perform an unnecessary action can even be considered an unexpected and negative behavior.^3^

Now, one may want to evaluate the truth value of the intuitive proposition p, and compare it with alternative hypotheses, to identify which is the most likely to be true. This questioning of one's intuitions requires some additional meta-cognitive efforts especially when it comes to scientific information, whose complexity makes it inaccessible to single individuals relying on their own knowledge.

Much work has looked into the reasons why people come to believe in fake news or conspiracy theories, especially since the beginning of the pandemic (van Mulukom et al., 2020). Of particular interest, the propensity to rely on analytical rather than intuitive thinking seems to be a strong predictor of adherence to epistemically questionable theories, including with respect to HCQ (Fuhrer and Cova, 2020; Pennycook and Rand, 2020). Both dispositional and contextual factors are most probably involved in the metacognitive ability to step back from one's own intuitions and to direct one's argumentative efforts in an accuracy-driven way. Personality traits such as impulsivity (Kuhn et al., 2021) and rigidity in one's belief structures (Meyer et al., 2021) have been linked to adherence to ill-founded ideas. Stressful environments (Swami et al., 2016) and the sense of lack of control (Whitson and Galinsky, 2008) are also likely to be favorable grounds for these beliefs.

It must also be emphasized that from an individual point of view, it is only worth engaging in reflective thinking about a theory if the stakes are high of knowing whether the theory is true. Only in such a case will additional (and truthful) information become necessary. Individuals' propensity to question their intuitions thus depends greatly on what the truth about a given subject means to them, compared to the value of maintaining and sharing these intuitions.

## 3. Low Stakes in Knowing the Truth

People's interest in the truth on a scientific matter can stem from their epistemic curiosity when prior knowledge already exists, allowing for relevant associations to be anticipated and knowledge gaps to be filled (Loewenstein, 1994; Litman, 2005). Accurate scientific information also matters when the accuracy of the knowledge on which people's decisions are based have tangible consequences for their own lives. False information may lead to harmful decisions, for instance when patients must make choices about treatments. We argue that the truth about the efficacy of HCQ was not “need to know” information. On the contrary, the stakes of knowing the truth about its actual effects on Covid-19 patients has remained relatively low at the individual level.

First, the extent to which the fear-inducing nature of the pandemic increased people's interest in being administered a truly efficient drug against Covid-19 is not evidenced in practice. At the beginning of 2020, the virus was said to be of little danger for healthy adults and children, and mainly lethal for elderly people with co-morbidity (Baud et al., 2020). It was even (falsely) compared to the seasonal flu at the beginning of the pandemic, including by Raoult and his team, as evidence of its harmlessness (Giraud-Gatineau et al., 2020). In contrast to the adverse effects of restrictive measures aimed at curbing the spread of the virus (such as wearing masks and locking down the economy, which were perceived by many as an unwarranted infringement of the people's freedom and well-being), the question of whether HCQ actually worked was ultimately of minor importance to most people.

Second, the risks associated with taking HCQ were from the outset perceived as very low. HCQ has been a well-known molecule for a long time, and Plaquenil could even be dispensed in pharmacies without a prescription before the pandemic.^4^ Moreover, many Covid-19 patients have testified about the harmless (and apparently effective) nature of the treatment. Only a few isolated accidents have been reported in the media, such as the American citizen who died after he and his wife ingested chloroquine phosphate, a product used to clean aquariums (Vigdor, 2020).

The practical benefits associated with knowing the truth about HCQ were thus small from an individual perspective. More generally, there are many aspects of our lives for which we are not accuracy-driven and many beliefs we entertain do not come under close scrutiny. In his veritistic framework, philosopher Alvin Goldman considered that a person's doxastic attitude toward a proposition p could not have a veritistic value if that person was uninterested in the question of p vs. not-p (). The dominance of relevance over truth has been discussed at length in the philosophical, epistemological, and pragmatic literature (Wilson and Sperber, 2002; Baumberger, 2014).

In sum, we are generally not epistemically committed to one particular side of a scientific dispute, and we live with many false or approximate beliefs (Boyer, 2018; Oliver and Wood, 2018). This is not a problem in itself, as long as these beliefs do not lead individuals to make choices against their own interests. Their lack of expertise becomes problematic when they are interested in defending a point of view on the topic at hand, for reasons that are unrelated to accuracy. As we shall see, people may find an interest in consuming and sharing ill-founded theories that they would be wary of—or even reject—in other circumstances, if social and emotional goals outweigh the stakes of knowing the truth.

## 4. Self-Relevant Beliefs about Raoult and HCQ

Information consumption is guided by the desire to maintain positive identities and coherent worldviews (Alicke et al., 2020). Coming up with an explanation that makes sense is satisfying, especially if it is not accessible to everyone (Lantian et al., 2017). In this respect, the literature on beliefs in conspiracy theories is illuminating. It shows that they respond to social-existential motives that individuals may have (van Mulukom et al., 2020). They foster a feeling of being able to understand the world from a global point of view rather than from a subjective and necessarily limited perspective. For instance, people are more likely to believe in conspiracy theories when they involve events that they feel the need to explain (Lantian et al., 2021), or target groups they perceive as culturally or politically threatening (Nera et al., 2021). In addition, people are more attracted to conspiracy theories when important psychological needs are frustrated, for instance when feeling socially excluded (Graeupner and Coman, 2017).

The transmission of relevant information is also an important way of manifesting one's competence and managing one's reputation (Boyer et al., 2015). Communication, including on science issues, may serve other purposes than informing, such as cultivating social relationships or demonstrating membership in a group (Baumeister and Leary, 1995; Dunbar, 2012; Mercier, 2020). When the objective moves away from the transmission of information per se, communication is no longer associated with a strong presumption of truthfulness (Lynch, 2004; Cassam, 2018). Besides, people do not always read what they share on social networks (Gabielkov et al., 2016), and there is a gap between what people actually read and what they share (Bright, 2016). The pragmatic meaning of a message then goes far beyond its literal interpretation (Grice, 1957). It becomes less about asserting that the proposition is true than conveying an attitude. This use of information as a mean to manage relations with one's ingroup is illustrated by recent works showing that anti-outgroup language is a strong predictor of social engagement on social media (Rathje et al., 2021), and that individuals who report hating their political opponents are the most likely to share political fake news (Osmundsen et al., 2021). This perspective implies that a major strength of questionable information and theories is the social success they enjoy, due to people's propensity to share them for other reasons than genuine belief in their accuracy.

In the case of HCQ, support for Raoult and his treatment resonated with the growing mistrust toward French institutions from a particular segment of the French population. In autumn 2019, the movement of the “Gilets Jaunes” began against green fuel taxes, and rapidly extended to a more general questioning of fiscal policy. It was fed by feelings that the government despised and was disconnected from the reality of the French middle class. Raoult's position against the “Parisian marquesses” echoed the movement's political fight against the elites, which are perceived as arrogant and disrespectful of popular practices and lifestyles (Sayare, 2020). The appeal to popular common sense and pragmatism, as opposed to experts suspected of denying reality, has been further used by politicians^5^ to justify value-based positions.

On April 6, 2020, a survey was released by French polling institute IFOP and published in the daily newspaper *Le Parisien*, asking citizens about their opinion on the efficacy of HCQ. The survey revealed that “59% of the French population believed HCQ was effective against the new coronavirus” (Mateus, 2020). Positive opinion was more frequent on the far right and far left, and reached 80% among sympathizers of the Gilets Jaunes. Support was also very high in the Marseille region, with a 74% rate of positive opinions. These results arguably reflect a positive attitude toward someone who stands against the current establishment rather than the outcome of truth-oriented reasoning. The analysis of the content of pro-Raoult pages on Facebook supports this interpretation (Audureau and Maad, 2020). The most frequently shared posts are not intended to convey (mis)information about HCQ, but to express one's adhesion to a particular value system, or simply to foster social interactions.^6^

## 5. Superficial opinions

Information need not be certain to be endorsed or shared, because part of what makes it valuable is not related to accuracy. In both cases, minimal plausibility can be enough, depending on what you—or your interlocutor—want to do with the information. One may endorse information, and defend it passionately, on the grounds that it is plausible, without trying to identify its actual probability of being true. Endorsing and sharing are intertwined to a certain point because people usually share information that they themselves endorse at least minimally, but they may sometimes have an interest in others' entertaining different beliefs. We avoid sharing information that could turn out to be blatantly false, but uncertain information can be highly relevant socially, and easily shared if the reputational costs, if ever it is fake, are not too high.

Because scientific information is complex, it is very difficult to find arguments that are intuitive enough to challenge it. Most scientific propositions are also not verifiable through simple perception and inference (Sperber, 1997), and individuals who are interested in having a good understanding of the state of a scientific question have no choice but to rely on experts. This cognitive opacity favors the strategic use of information for non-epistemic purposes, and leaves room for motivated reasoning, that is, the processing of arguments in a selective way, aimed at defending self-relevant beliefs, rather than forming an accurate representation of reality (Kunda, 1990; Kahan, 2016). Cognitive effort and the search for new arguments are then preferentially directed toward the justification of one's epistemic position (Mercier and Sperber, 2011).

For example, if the value of promoting HCQ is unrelated to its actual effectiveness, Raoult's supporters will be less motivated to direct their reasoning efforts toward finding the truth about it. Instead, supporters will be highly motivated to gather arguments likely to justify their position. Integrating more elaborate considerations such as the specific effect of combining HCQ with azithromycin implies some reasoning effort, but this effort is arguably not directed toward searching for truth.

Decreasing social-existential needs makes people less eager to rely on motivated reasoning. For instance, Nyhan and Reifler (2019) have found that conservatives were more likely to accept facts and arguments about withdrawing the military from Iraq in a situation where self-esteem was experimentally enhanced. Conversely, when the stakes of holding certain beliefs as true are too important, people may refuse the evidence even though it would allow them to make better decisions for themselves or their loved ones. We suggest that social-existential stakes can be powerful enough to outweigh the usefulness of true information.

## 6. Societal Implications

The implications of scientific information can be quite different at the individual and societal level. As we have seen, from the individual's point of view, scientific knowledge is useful to the general public up to a certain point. Depending on what one wants to do with scientific information, an accurate representation of the world can be of paramount importance, or merely the icing on the cake.

Conversely, the success of a scientific endeavor at the societal level depends on its ability to build a fair representation of the true state of the world, and it has major consequences when it determines the outcome of decisions taken by society as a whole. Building a science-based common ground shared by all the actors of a society is paramount to creating the conditions for this knowledge to be trusted and successfully translated into effective behavior. Crucially, publicity of public opinions may have performative effects, even when it does not reflect genuine adherence to the assumptions on which people are being asked to give their views. When these opinions enter the public sphere, they influence the way societal issues are conceived, and patterns of thinking that are coalitional rather than collaborative can become dominant in the public debate. This can in turn negatively affect the quality of the political decisions that are made and that will have concrete consequences for the well-being of the populations.

Regarding the case of HCQ, it is not certain that the results of the IFOP survey published in *Le Parisien* would have been the same in a situation where the stakes of giving a correct answer had been high. But the media coverage of these opinions turned out to have quite real political and social consequences. Politics attended to particular voices who would have been isolated otherwise. For example, Emmanuel Macron visited the IHU Méditerranée Infection at the beginning of April 2020 as a way to establish a dialogue with a distrustful population. A close collaborator of Emmanuel Macron testified that the president could not stay away from the “Raoult effect” for too long (*Le Monde*, April 9, 2020). In doing so, he was acting upon a public opinion that did not reflect a strong epistemic commitment as opposed to prioritizing the scientific consensus of the time. Furthermore, media coverage of this controversy had the effect of delaying clinical trials, especially with respect to the European Discovery project which had subsequent difficulties recruiting patients for experimental groups other than those testing HCQ (Tikkinen et al., 2020). It has also caused supply problems in pharmacies, with consequences for patients for whom this treatment was necessary.

Public opinions on scientific issues must thus be interpreted at the right level. Otherwise, positions defended for social or identity-related reasons can lead to the undermining of the scientific common ground on which decisions taken at societal level are based. If this common ground is not clearly identified and shared by all, public debate becomes biased when instead it deserves important efforts of collaborative reasoning. This is all the more necessary when it comes to health issues that have political significance, from vaccines to protective measures in the context of a pandemic.

## 7. Conclusion

The case of HCQ illustrates the importance of better understanding people's relationship to scientific information. People share opinions everyday for a variety of reasons that go beyond the mere transmission of accurate information. This is not necessarily a problem in itself, but when these beliefs occupy the public space, they can end up having harmful societal consequences, especially when it comes to health issues. As in the HCQ case, individuals may not have any interest in knowing once and for all whether a drug has any utility at all, yet still publicly express their opinion on the issue. The question of how to interpret these publicly held beliefs is therefore important, both from a theoretical and a practical point of view, and constitutes an exciting field of research with important political implications.

## Data Availability Statement

The original contributions presented in the study are included in the article/supplementary material, further inquiries can be directed to the corresponding author/s.

## Author Contributions

TM wrote the first version of the manuscript with support from TYB and GO. All authors contributed to its final version.

## Funding

This work was supported by the European Union's Horizon 2020 project under Grant number 870883—PEriTiA.

## Conflict of Interest

TM was employed by Strane Innovation. The remaining authors declare that the research was conducted in the absence of any commercial or financial relationships that could be construed as a potential conflict of interest.

## Publisher's Note

All claims expressed in this article are solely those of the authors and do not necessarily represent those of their affiliated organizations, or those of the publisher, the editors and the reviewers. Any product that may be evaluated in this article, or claim that may be made by its manufacturer, is not guaranteed or endorsed by the publisher.

## Footnotes

^1^See retroactive statement from the International Society of Antimicrobial Chemotherapy (ISAC) stating that “the article does not meet the Society's expected standard, especially relating to the lack of better explanations of the inclusion criteria and the triage of patients to ensure patient safety” (International Society of Antimicrobial Chemotherapy, 2020).

^2^Raoult's position on this issue has not changed: on February 11, 2021, he published *Carnets de guerre Covid-19*, in which he collected his writings and interventions on the YouTube channel of his institution.

^3^For example, Wallis et al. (2017) suggest that doctors find it hard to stop prescriptions when they become unnecessary for fear of being seen as bad doctors.

^4^Plaquenil has been subject to medical prescription in France since January 2020.

^5^For instance, in an interview with the magazine *L'Express* on May 18, 2021, the French Minister of the Interior Gérald Darmanin said that he preferred the “common sense of the butcher of Tourcoing” to the studies of the National Institute of Statistics and Economic Studies.

^6^See also Berriche and Altay (2020) who highlighted the prevalence of phatic posts in the pseudoscientific Facebook page *Santé + Mag*.

## References

[B1] Agoritsas T. Boroli F. Calmy A. Gartner B. Gasche P. Gayet-Ageron A. (2020). Chloroquine, Hydroxychloroquine et COVID-19: Evaluation Pharmacologique. Hôpitaux Universitaires de Genève. Available online at: https://www.hug.ch/sites/interhug/files/structures/coronavirus/documents/hydroxy-chloroquine-et-covid-19.pdf

[B2] Alicke M. D. Sedikides C. Zhang Y. (2020). The motivation to maintain favorable identities. Self Ident. 19, 572–589. 10.1080/15298868.2019.1640786

[B3] Audureau W. Maad A. (2020). Une exploration de la “Raoultsphére” sur Facebook. Le Monde. Available online at: https://www.lemonde.fr/les-decodeurs/article/2020/07/03/une-exploration-de-la-raoultsphere-sur-facebook_6045017_4355770.html (accessed July 3, 2020)

[B4] Baud D. Qi X. Nielsen-Saines K. Musso D. Pomar L. Favre G. (2020). Real estimates of mortality following COVID-19 infection. Lancet Infect. Dis. 20:773. 10.1016/S1473-3099(20)30195-X 32171390 PMC7118515

[B5] Baumberger C. (2014). Art and understanding. Defence of Aesthetic Cognitivism, in Bilder Sehen Perspektiven der Bildwissenschaft, eds Greenlee M. Hammwohner R. Köber B. Wagner C. Wolff C. (Regensburg, Schnell und Steiner), 1–24.

[B6] Baumeister R. F. Leary M. R. (1995). The need to belong: desire for interpersonal attachments as a fundamental human motivation. Psychol. Bull. 117:497. 10.1037/0033-2909.117.3.497 7777651

[B7] Beck J. Forstmeier W. (2007). Superstition and belief as inevitable by-products of an adaptive learning strategy. Hum. Nat. 18, 35–46. 10.1007/BF02820845 26181743

[B8] Berriche M. Altay S. (2020). Internet users engage more with phatic posts than with health misinformation on Facebook. Palgrave Commun. 6, 1–9. 10.1057/s41599-020-0452-1

[B9] Bik E. (2020). Thoughts on the Gautret et al. paper about hydroxychloroquine and Azithromycin treatment of COVID-19 infections. Sci. Integr. Digest, 24. Available online at: https://scienceintegritydigest.com/2020/03/24/thoughts-on-the-gautret-et-al-paper-about-hydroxychloroquine-and-azithromycin-treatment-of-covid-19-infections/

[B10] Boyer P. (2018). Minds Make Societies: How Cognition Explains the World Humans Create. Yale University Press.

[B11] Boyer P. Firat R. van Leeuwen F. (2015). Safety, threat, and stress in intergroup relations: a coalitional index model. Perspect. Psychol. Sci. 10, 434–450. 10.1177/1745691615583133 26177946

[B12] Branch T. Origgi G. Morisseau T. (2021). Trust, expertise and the controversy over hydroxychloroquine. Soc. Epistemol. Available online at: https://hal.archives-ouvertes.fr/hal-03095293. [Epub ahead of print].

[B13] Bright J. (2016). The social news gap: how news reading and news sharing diverge. J. Commun. 66, 343–365. 10.1111/jcom.12232

[B14] Cassam Q. (2018). Epistemic insouciance. J. Philos. Res. 43, 1–20. 10.5840/jpr2018828131

[B15] Damisch L. Stoberock B. Mussweiler T. (2010). Keep your fingers crossed! How superstition improves performance. Psychol. Sci. 21, 1014–1020. 10.1177/0956797610372631 20511389

[B16] Dunbar R. I. (2012). Bridging the bonding gap: the transition from primates to humans. Philos. Trans. R. Soc. B Biol. Sci. 367, 1837–1846. 10.1098/rstb.2011.0217 22641822 PMC3367699

[B17] EMA (2020). COVID 19: Chloroquine and Hydroxychloroquine Only To Be Used in Clinical Trials or Emergency Use Programmes. Amsterdam: EMA.

[B18] Foster K. R. Kokko H. (2009). The evolution of superstitious and superstition-like behaviour. Proc. R. Soc. B Biol. Sci. 276, 31–37. 10.1098/rspb.2008.0981 18782752 PMC2615824

[B19] Fuhrer J. Cova F. (2020). Quick and dirty”: intuitive cognitive style predicts trust in Didier Raoult and his hydroxychloroquine-based treatment against COVID-19. Judgm. Decis. Mak. 15, 889–908. Available online at: http://journal.sjdm.org/20/200731/jdm200731.html

[B20] Gabielkov M. Ramachandran A. Chaintreau A. Legout A. (2016). Social clicks: what and who gets read on twitter? SIGMETRICS Perform. Eval. Rev. 44, 179–192. 10.1145/2964791.2901462

[B21] Gautret P. Lagier J.-C. Parola P. Meddeb L. Mailhe M. Doudier B.. (2020). Hydroxychloroquine and azithromycin as a treatment of COVID-19: results of an open-label non-randomized clinical trial. Int. J. Antimicrob. Agents 56:105949. 10.1016/j.ijantimicag.2020.105949 32205204 PMC7102549

[B22] Giraud-Gatineau A. Colson P. Jimeno M.-T. Zandotti C. Ninove L. Boschi C.. (2020). Comparison of mortality associated with respiratory viral infections between December 2019 and March 2020 with that of the previous year in Southeastern France. Int. J. Infect. Dis. 96, 154–156. 10.1016/j.ijid.2020.05.001 32389848 PMC7204704

[B23] Goldman A. (2002). Knowledge in a social world. Phil. Phenomenol. Res. 64, 185–190. 10.1111/j.1933-1592.2002.tb00151.x

[B24] Graeupner D. Coman A. (2017). The dark side of meaning-making: how social exclusion leads to superstitious thinking. J. Exp. Soc. Psychol. 69, 218–222. 10.1016/j.jesp.2016.10.003

[B25] Grice H. P. (1957). Meaning. Philos. Rev. 66, 377–388. 10.2307/2182440

[B26] International Society of Antimicrobial Chemotherapy (2020). Official Statement From International Society of Antimicrobial Chemotherapy (ISAC). International Society of Antimicrobial Chemotherapy.

[B27] Kahan D. M. (2016). The politically motivated reasoning paradigm, Part 1: What politically motivated reasoning is and how to measure it”, in Emerging Trends in the Social and Behavioral Sciences: An Interdisciplinary, Searchable, and Linkable Resource, eds Scott R. A. Kosslyn S. M. (John Wiley & Sons), 1–16.

[B28] Kuhn S. A. K. Lieb R. Freeman D. Andreou C. Zander-Schellenberg T. (2021). Coronavirus conspiracy beliefs in the German-speaking general population: endorsement rates and links to reasoning biases and paranoia. Psychol. Med. 1–15. 10.1017/S0033291721001124 33722315 PMC8027560

[B29] Kunda Z. (1990). The case for motivated reasoning. Psychol. Bull. 108:480. 10.1037/0033-2909.108.3.480 2270237

[B30] Lamontagne F. Agoritsas T. Siemieniuk R. Rochwerg B. Bartoszko J. Askie L.. (2021). A living who guideline on drugs to prevent COVID-19. BMJ 372:n526. 10.1136/bmj.n526 33649077

[B31] Lantian A. Bagneux V. Delouvée S. Gauvrit N. (2021). Maybe a free thinker but not a critical one: High conspiracy belief is associated with low critical thinking ability. Appl. Cogn. Psychol. 35, 674–684. 10.1002/acp.3790

[B32] Lantian A. Muller D. Nurra C. Douglas K. M. (2017). I know things they don't know! Soc. Psychol. 48, 160–173. 10.1027/1864-9335/a000306

[B33] Litman J. (2005). Curiosity and the pleasures of learning: wanting and liking new information. Cogn. Emot. 19, 793–814. 10.1080/02699930541000101

[B34] Liu J. Cao R. Xu M. Wang X. Zhang H. Hu H.. (2020). Hydroxychloroquine, a less toxic derivative of chloroquine, is effective in inhibiting SARS-COV-2 infection *in vitro*. Cell Discov. 6, 1–4. 10.1038/s41421-019-0132-8 32194981 PMC7078228

[B35] Loewenstein G. (1994). The psychology of curiosity: a review and reinterpretation. Psychol. Bull. 116:75. 10.1037/0033-2909.116.1.75

[B36] Lynch M. P. (2004). Who cares about the truth? Chronicle High. Educ. 51:B6. Available online at: https://www.chronicle.com/article/who-cares-about-the-truth/

[B37] Mateus C. (2020). COVID-19: 59% des Français Croient á l'efficacité de la Chloroquine. Le Parisien. Available online at: http://www.leparisien.fr/societe/sante/covid-19-59-des-francais-croient-a-l-efficacite-de-la-chloroquine-05-04-2020-8294535.php

[B38] Mercier H. (2020). Not Born Yesterday. Princeton, NJ: Princeton University Press. 10.1515/9780691198842

[B39] Mercier H. Sperber D. (2011). Why do humans reason? Arguments for an argumentative theory. Behav. Brain Sci. 34, 57–74. 10.1017/S0140525X10000968 21447233

[B40] Meyer M. Alfano M. De Bruin B. (2021). Epistemic vice predicts acceptance of Covid-19 misinformation. Episteme, 1–22. 10.1017/epi.2021.18

[B41] Nera K. Wagner-Egger P. Bertin P. Douglas K. Klein O. (2021). A power-challenging theory of society, or a conservative mindset? Upward and downward conspiracy theories as ideologically distinct beliefs. Eur. J. Soc. Psychol. 10.1002/ejsp.2769. [Epub ahead of print].

[B42] Nyhan B. Reifler J. (2019). The roles of information deficits and identity threat in the prevalence of misperceptions. J. Elect. Publ. Opin. Part. 29, 222–244. 10.1080/17457289.2018.1465061

[B43] Oliver J. E. Wood T. J. (2018). Enchanted America: How Intuition and Reason Divide Our Politics. Chicago, IL: University of Chicago Press. 10.7208/chicago/9780226578644.001.0001

[B44] Osmundsen M. Bor A. Vahlstrup P. B. Bechmann A. Petersen M. B. (2021). Partisan polarization is the primary psychological motivation behind political fake news sharing on Twitter. Am. Polit. Sci. Rev. 115, 999–1015. 10.1017/S0003055421000290

[B45] Pennycook G. Rand D. G. (2020). Who falls for fake news? The roles of bullshit receptivity, overclaiming, familiarity, and analytic thinking. J. Pers. 88, 185–200. 10.1111/jopy.12476 30929263

[B46] Rathje S. Van Bavel J. J. van der Linden S. (2021). Out-group animosity drives engagement on social media. Proc. Natl. Acad. Sci. U.S.A. 118:e2024292118. 10.1073/pnas.2024292118 34162706 PMC8256037

[B47] Risch H. (2020). The key to defeating COVID-19 already exists. We need to start using it. Newsweek August 22:2020. Available online at: https://www.newsweek.com/key-defeating-covid-19-already-exists-we-need-start-using-it-opinion-1519535

[B48] Sauvayre R. (2020). Retour sur le débat médiatique et éthique concernant le traitement contre la covid-19 á base de chloroquine. Les Cahiers de l'Espace Ethique, 91–92.

[B49] Sayare S. (2020). He Was a Science Star. Then He Promoted a Questionable Cure for Covid-19. The New York Times. Available online at: https://www.nytimes.com/2020/05/12/magazine/didier-raoult-hydroxychloroquine.html

[B50] Sciama Y. (2020). Is France's president fueling the hype over an unproven coronavirus treatment. Science. 10.1126/science.abc1786

[B51] Shermer M. (1997). Why People Believe Weird Things: Pseudoscience, Superstition, and Other Confusions of Our Time. New York, NY: Henry Holt and Company.

[B52] Solomon V. R. Lee H. (2009). Chloroquine and its analogs: a new promise of an old drug for effective and safe cancer therapies. Eur. J. Pharmacol. 625, 220–233. 10.1016/j.ejphar.2009.06.063 19836374

[B53] Sperber D. (1997). Intuitive and reflective beliefs. Mind Lang. 12, 67–83. 10.1111/1468-0017.00036

[B54] Swami V. Furnham A. Smyth N. Weis L. Lay A. Clow A. (2016). Putting the stress on conspiracy theories: examining associations between psychological stress, anxiety, and belief in conspiracy theories. Pers. Individ. Differ. 99, 72–76. 10.1016/j.paid.2016.04.084

[B55] Tikkinen K. A. Malekzadeh R. Schlegel M. Rutanen J. Glasziou P. (2020). COVID-19 clinical trials: learning from exceptions in the research chaos. Nat. Med. 26, 1671–1672. 10.1038/s41591-020-1077-z 32963376

[B56] van Mulukom V. Pummerer L. Alper S. Bai H. Cavojova V. Farias J.. (2020). Antecedents and consequences of Covid-19 conspiracy beliefs: A systematic review. PsyarXiv [Preprint]. 10.31234/osf.io/u8yah PMC8920084 35354105

[B57] Vigdor N. (2020). Man fatally poisons himself while self-medicating for coronavirus, doctor says. New York Times 24.

[B58] Wallis K. A. Andrews A. Henderson M. (2017). Swimming against the tide: primary care physicians' views on deprescribing in everyday practice. Ann. Fam. Med. 15, 341–346. 10.1370/afm.2094 28694270 PMC5505453

[B59] Whitson J. A. Galinsky A. D. (2008). Lacking control increases illusory pattern perception. Science 322, 115–117. 10.1126/science.1159845 18832647

[B60] WHO (2020). Who Director-General's Opening Remarks at the Media Briefing on COVID-19 - 11 March 2020. WHO.

[B61] WHO Solidarity Trial Consortium (2021). Repurposed antiviral drugs for COVID-19-interim who solidarity trial results. N. Engl. J. Med. 384, 497–511. 10.1056/NEJMoa2023184 33264556 PMC7727327

[B62] Wilson D. Sperber D. (2002). Truthfulness and relevance. Mind 111, 583–632. 10.1093/mind/111.443.583

